# Droplet Microfluidics-Enabled High-Throughput Screening for Protein Engineering

**DOI:** 10.3390/mi10110734

**Published:** 2019-10-29

**Authors:** Lindong Weng, James E. Spoonamore

**Affiliations:** enEvolv, Inc., Medford, MA 02155, USA; j.spoonamore@enevolv.com

**Keywords:** FADS, emulsification, droplet coalescence, enzyme engineering, synthetic biology

## Abstract

Protein engineering—the process of developing useful or valuable proteins—has successfully created a wide range of proteins tailored to specific agricultural, industrial, and biomedical applications. Protein engineering may rely on rational techniques informed by structural models, phylogenic information, or computational methods or it may rely upon random techniques such as chemical mutation, DNA shuffling, error prone polymerase chain reaction (PCR), etc. The increasing capabilities of rational protein design coupled to the rapid production of large variant libraries have seriously challenged the capacity of traditional screening and selection techniques. Similarly, random approaches based on directed evolution, which relies on the Darwinian principles of mutation and selection to steer proteins toward desired traits, also requires the screening of very large libraries of mutants to be truly effective. For either rational or random approaches, the highest possible screening throughput facilitates efficient protein engineering strategies. In the last decade, high-throughput screening (HTS) for protein engineering has been leveraging the emerging technologies of droplet microfluidics. Droplet microfluidics, featuring controlled formation and manipulation of nano- to femtoliter droplets of one fluid phase in another, has presented a new paradigm for screening, providing increased throughput, reduced reagent volume, and scalability. We review here the recent droplet microfluidics-based HTS systems developed for protein engineering, particularly directed evolution. The current review can also serve as a tutorial guide for protein engineers and molecular biologists who need a droplet microfluidics-based HTS system for their specific applications but may not have prior knowledge about microfluidics. In the end, several challenges and opportunities are identified to motivate the continued innovation of microfluidics with implications for protein engineering.

## 1. Introduction

Engineered proteins with tailored properties evolved from natural precursors have been playing an increasingly important role in a spectrum of agricultural, industrial, and biomedical applications [1,2]. For example, protein engineering holds the potential of transforming the metabolic drug landscape through the development of smart, stimulus-responsive drug systems [3]. In industrial processes, protein engineering enables the production of enzymes that offer unique advantages compared with chemical catalysts, such as biodegradability, stereoselectivity, substrate specificity, functionality under relatively mild solvents, temperatures, pHs and pressures, and stability at extreme conditions [4,5]. Today, protein engineering also plays a critical role in advancing the emerging field of synthetic biology, including optimizing pathway enzymes and regulatory elements, balancing pathway redox equivalents, as well as tuning the expression of pathway genes.

There are two main strategies in protein engineering: rational design and directed evolution. Rational design, which is mostly carried out in silico, is knowledge-based, deterministic engineering of proteins. Thus, it needs prior information on the target protein such as a structural model, sequence relationship to homologs, and insights into its biophysical function. As a powerful approach to test general theories in protein chemistry, rational design can be achieved either by single-point mutation, exchange of elements of secondary structure, exchange of whole domains, or by fusion of enzymes. Novel computational tools have constantly improved, resulting in dramatic increase in the sizes of mutant libraries that can be designed. Genome-scale metabolic models (GEMs) have informed and expanded variant design for many industrially relevant microorganisms [6]. Furthermore, multiplex automated genome evolution (MAGE) [7] and CRISPR/Cas [8,9] systems have significantly improved the throughput of genome editing with precision and reduced cost and time required to explore many protein targets. For example, MAGE enables the rapid generation of billions of mutants by repeated insertion, deletion or mutation of DNA at multiple chromosomal targets [7]. Overall, the unprecedented capability of designing and building a large number of variants for rational protein design has placed increased demands on the throughput of screening and selection [10,11].

Directed evolution, on the other hand, mimics the process of natural selection through random mutagenesis to steer proteins or nucleic acids toward desired traits [12,13,14]. Unlike rational protein design, directed evolution requires neither prior knowledge of a protein’s detailed structure nor prediction of the effects of various mutations [15]. Indeed, directed evolution makes it possible to identify undiscovered protein sequences which have novel functions. Moreover, synthetic biologists also increasingly rely on directed evolution to optimize engineered biological systems [16,17,18]. However, for directed evolution to be truly effective, very large libraries of mutants must be screened under conditions that closely match the desired functionality [19]. Thus, the throughput of screening for variants with improved traits is a major factor dictating the efficiency of directed evolution given that the libraries of random mutants can be easily on the scale of 10^8^–10^9^ [19,20,21].

Proteins are engineered for properties such as affinity, selectivity, stability or enzymatic activity [22]. Often, when a cell based fluorescent readout is achievable, screening of engineered protein libraries is performed with fluorescence-activated cell sorting (FACS) to separate a population of cells into sub-populations based on fluorescent labeling [22]. In this case, the phenotype-genotype connection is unbroken because the cells are selected directly. For example, Lipovšek et al. [23] reported in vitro selection of catalytically active enzymes (horseradish peroxidase) from large libraries of variants displayed on the surface of the yeast *S. cerevisiae* and separated by FACS. To improve substrate specificity of glycosyltransferases by directed evolution, Aharoni et al. [24] screened a library of over a million sialyltransferases mutants using FACS and found a variant with up to 400-fold higher catalytic efficiency for transfer to a variety of fluorescently labeled acceptor sugars. In their study, the formation of sialosides in intact *E. coli* cells was detected by selectively trapping the fluorescently labeled transfer products within the cell and the resulting cell population was analyzed and sorted using FACS [24]. However, many desirable properties are not amenable to direct interrogation via FACS because the phenotype is not inherent in a single cell, for example, when improving a protein excreted into growth medium. Properties such as extracellular analyte consumption, product secretion and cell-cell interactions are not readily detectable with flow cytometry. Screening for ‘non-cellular’ phenotypes necessitates the compartmentalization of single cells or an alternative expression system to maintain the linkage between the phenotype that the selection acts on and the genotype in which the evolutionary information is encoded [19]. Compartmentalization of assays in arrays of wells makes microtiter plates by far the most widely used screening platform. However, the microplate-based method becomes problematic when the assay volume is less than 1 µL due to evaporation and capillary forces [25]. Even with robotic automation for liquid handling using 1536-well plates and assuming a processing rate of 1 plate per minute, the throughput of a well based assay is approximately 25 samples/sec for a prompt optical measurement.

Miniaturization of screening systems can substantially increase sorting efficiency, improve selection and reduce screening costs, enabling exploration of very large libraries (10^8^–10^9^). These advantageous properties have stimulated emerging micro- and nanotechnologies to move into applications in the life sciences and molecular biology. Early efforts included the in vitro compartmentalization demonstrated by Tawfik and Griffiths [26] in late 1990s. They showed the selection of genes encoding HaeIII methyltransferase from a 10^7^-fold excess of genes encoding another enzyme using water-in-oil emulsions. These polydisperse droplets were generated by adding an in vitro transcription/translation reaction mixture into stirred mineral oil containing surfactants. As many assays require an accurate and reliable means of fluid manipulation to enable reproducible results, polydispserse droplets can be problematic. In the sub-microliter or sub-nanoliter volume range, droplet microfluidics, which emerged at the beginning of 2000s [27], presented a new paradigm for screening, offering precise and reduced reagent volumes as well as single-cell resolution analysis [28]. Microfluidic devices, featuring a network of channels with dimensions from tens to hundreds of micrometers, enable the generation and digital manipulation of droplets of uniform sizes (microliter to femtoliter) at very high throughput (up to several kHz). Surfactant systems enable the stabilization of droplets such that they can be incubated off-chip and reintroduced intact into subsequent microfluidic device(s) for sorting and analysis. However, it was not until the most recent decade that the HTS capacity of droplet microfluidics had been demonstrated for protein engineering, especially directed evolution [19,21,22,29,30,31].

**Figure 1:**
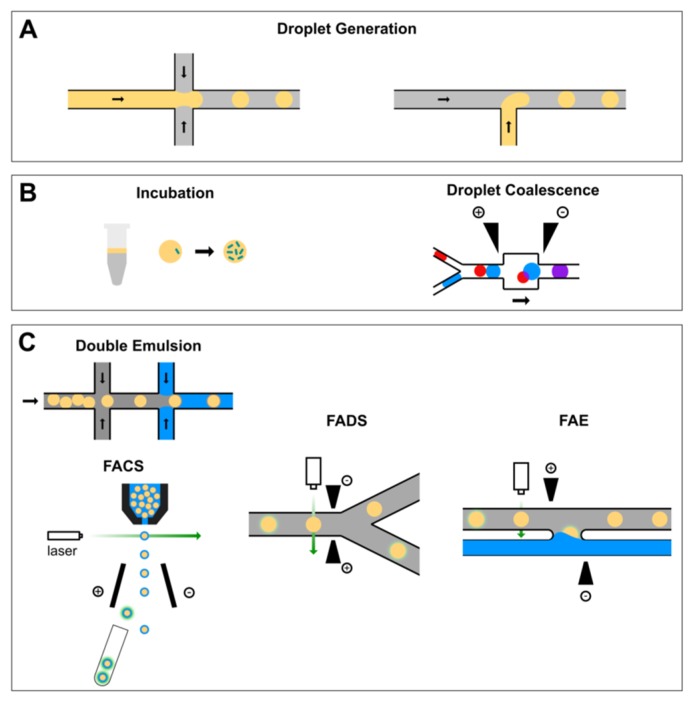
A typical workflow of droplet-based high-throughput screening system for protein engineering. (**A**) Single cells are encapsulated into monodisperse water-in-oil droplets generated on a flow-focusing (left) or T-junction (right) nozzle. (**B**) Cell-laden droplets can be incubated either on-chip or off-chip and new reagents can be added via droplet coalescence. (**C**) Droplets of interest can be sorted either by fluorescence-activated cell sorting (FACS) after an extra step of double emulsification or by fluorescence-activated droplet sorting (FADS). Alternatively, the content of droplets of interest can be recovered via fluorescence-activated electrocoalescence (FAE).

Here, we review the recent high-throughput screening systems developed for protein engineering that are enabled by droplet microfluidics. The review follows the structure of a typical workflow, as illustrated in Figure 1, which includes the following modules: emulsification, incubation, reagent addition, and sorting. This review article can also serve as a tutorial guide for those who need a droplet-based HTS system for their specific applications but may not have prior knowledge about microfluidics. A number of key challenges and opportunities are outlined in the end to motivate the continued innovation of microfluidics with implications for protein engineering.

## 2. Droplet-Based Hight-Throughput Screening

### 2.1. Microfluidics Droplet Generation

Droplet formation in microfluidic devices utilizes flows of two immiscible liquids that are controlled independently [27]. Most commonly, an aqueous solution is dispersed into a continuous phase of oil. The T-junction and flow-focusing nozzle (Figure 1A) are the most widely adopted geometries of microfluidic channels for generating droplets, where the stream of the dispersed fluid is pinched off by the continuous carrier fluid through shearing to generate monodisperse droplets. The frequency of droplet generation can reach up to 10 kHz and the droplets volumes may range from femtoliters to nanoliters. There have been several reviews available in the literature that provide complete details about droplet generation using microfluidics [22,32,33,34,35]. Therefore, the current review only discusses several fundamental elements in droplet generation using microfluidics, in particular, formulations (i.e., oils and surfactants).

As self-contained microreactors, monodisperse water-in-oil droplets have been used predominantly in the miniaturization of biochemical assays. In this scenario, fluorinated oils such as HFE-7500 paired with fluorosurfactants are widely used as the continuous phase [35]. Fluorinated oils provide desirable merits such as low leakage of organic solutes, high solubility of respiratory gases, as well as compatibility with polydimethylsiloxane (PDMS)—one of the most important materials for flow delivery in microfluidic devices. Alternative systems based on hydrocarbon oils, such as mineral oil, have also been successfully used for applications such as PCR [36] and directed evolution of enzymes [37]. In hydrocarbon systems, however, hydrophobic solutes tend to phase partition into the oil which leads to the cross-contamination among droplets, thereby limiting the range of their applications [38]. It is worth mentioning that molecular diffusion is not necessarily excluded in fluorocarbon systems. For example, the exchange of certain fluorophores was also observed even in fluorinated oil (HFE-7500) [39].

The dispersion of an immiscible fluid into another creates a non-equilibrium thermodynamic system [38,40]. The addition of surfactants acts against the homogenization of each fluid in the system by providing an energy barrier for droplet coalescence, yielding a stabilized dispersion in a metastable state [38]. Although fluorinated oils have favorable properties for biochemical assays, only a limited number of surfactants are available for the stabilization of water-oil interfaces in these systems [38]. Perfluoropolyether-polyethylene glycol (PFPE-PEG), a triblock copolymer consisting of fluorinated alkyl domains, is the most commonly used surfactant in fluorinated oils. Its PEG domains are also critical to prevent nonspecific adsorption of proteins, DNA and RNA at the droplet interface. Surfactants compatible with hydrocarbons include sorbitan monooleate (Span 80), silicone-based ABIL EM 90/180, TWEEN 20/80 and so forth.

The oil-surfactant system has to be formulated carefully since it in part determines the capability of microfluidics to generate controlled liquid structures with complex functionalities at the interface for a variety of applications including protein or enzyme engineering. When selecting the combination of oil and surfactant, several factors need to be taken into consideration, including aqueous composition, biocompatibility, molecular exchange between droplets, stability (e.g., tolerance with thermocycling), as well as compatibility with double or multiple emulsification. Table 1 listed the formulations that were frequently used by the droplet-based screening studies discussed in the following sections.

### 2.2. Reagent Addition in Droplet Flow

Adding reagents into droplets is one of the most important functions in droplet-based microfluidic systems. Additions enable complex biochemical assays, for example, performing multistep reactions that require mixing new reagents at different times [41]. Reagent addition in droplet flow can be achieved by a variety of methods such as droplet coalescence induced by either electric field [42,43], physical constrictions [44], or certain chemicals [45].

**Figure 2:**
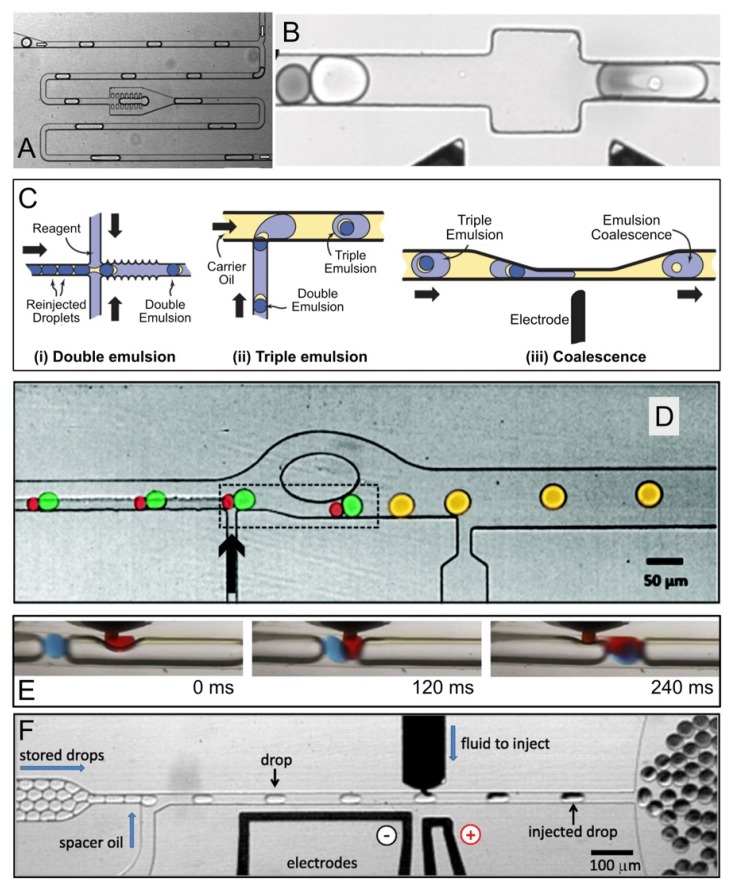
Droplet coalescence approaches. (**A**) The coalescence of two droplets within a pillar constriction-assisted droplet-merging device. Figure 2A was adapted from reference [44] with permission from the Royal Society of Chemistry. (**B**) A fusion module that delivered an alternating current (AC) field permitted electrically controlled merging of pairs of dye-containing droplets and cell-containing droplets. Figure 2B was adapted from reference [60], complying with the License for PNAS Articles. (**C**) Schematic of a different electrocoalescence workflow assisted with triple emulsification. At step (i), reinjected droplets are enveloped by an aqueous reagent phase in a hydrophilic channel. The resulting double emulsion travels to a hydrophobic junction at step (ii) where carrier oil encapsulates it to form a triple emulsion. At step (iii), the encapsulated double emulsion is ruptured in the presence of an electric field. Figure 2C was adapted from reference [50] with permission, copyright American Institute of Physics. (**D**) Passive microfluidic droplet coalescence through the addition of a destabilizing alcohol. Perfluorobutanol is added through the channel indicated by the black arrow, causing downstream coalescence of paired droplets. Droplets are false colored. Figure 2D was adapted from reference [45] with permission from the Royal Society of Chemistry. (**E**) A sequence of still images showing the direct injection of red-dyed octadecene into blue-dyed octadecene droplets, using Ar gas as a spacer and PFPE as the carrier fluid. Figure 2E was adapted from reference [61] under the Creative Commons Attribution license. (**F**) A picoinjector microfluidic device. The spacer adds oil from a side channel to space the droplets. The picoinjector injects fluid by merging the droplets with a pressurized channel containing the reagent. Picoinjection is triggered by an electric field, which is applied by the electrodes. Figure 2F was adapted from reference [41], complying with the License for PNAS Articles.

Niu et al. [44] developed a physical method to merge aqueous droplets within a microfluidic chamber featuring several rows of pillars separated by distances smaller than the representative droplet dimension. In a typical case (Figure 2A), a droplet entered the chamber, slowed down and stopped. This droplet would then merge with another droplet behind it when the surface tension was overwhelmed by the hydraulic pressure. It should be noted that in the above study hexadecane was used as the continuous phase without the addition of surfactants. Therefore, the efficiency of this physical coalescence method is subjected to validation for other oil/surfactant formulations.

Electrocoalescence is commonly used to merge pairs of droplets that are synchronized by size-dependent flow in microfluidic channels [42,49,60]. Two streams of different-sized droplets made independently (allowing different time scales, sizes, and compositions) can be synchronized in a single microfluidic channel. Smaller droplets move faster than larger droplets due to the Poiseuille flow. Therefore, the smaller droplets are brought into contact with preceding larger droplets (Figure 2B). Pairs of surfactant-stabilized droplets are coalesced while passing by an electric field. It was found that an electric field that was aligned with the flow direction could induce the maximum force to deform the adjacent surfaces of the paired droplets, thereby maximizing the effectiveness of electrocoalescence [62]. Alternatively, Sciambi and Abate [50] presented another method to add reagent to droplets by enveloping them as double emulsions in reagent-filled droplets and then rupturing the inner droplet with an electric field (Figure 2C). When the double emulsions ruptured, they released their contents into the enveloping droplets, ensuring mixing with reagent while limiting cross-droplet contamination.

In addition, Akartuna et al. [45] presented a chemically-mediated approach to coalesce pairs of surfactant-stabilized water-in-fluorocarbon oil droplets in a continuous flow (Figure 2D). Pairs of large and small droplets were exposed to perfluorobutanol, a poor solvent for the surfactant, before entering into a constriction channel. In the presence of perfluorobutanol, the water-oil interfaces became highly prone to coalescence due to the local depletion of surfactant. When the pairs exited the constriction, the velocity of the leading large droplet rapidly decreased, forcing the droplet pairs to contact and coalesce before re-stabilization. Overall, this coalescence method was able to perform ~300 droplet-merging events per second on a par with the rates of electrocoalescence methods [19,42,60], although it may be majorly limited by upstream pairing.

The coalescence-based methods discussed above involve an additional stream of droplets which encapsulate the reagent(s) to be added. The reagent droplets have to be synchronized with the target droplets such that they can be brought to close contact in pairs and merged via constriction-, chemical- or electric field-induced coalescence. The major advantage of coalescence-based methods is a broad range of reagent volume that can be added, especially for large-volume addition. Electrocoalescence also works compatibly with stable emulsions. On the downside, these methods, which rely on droplet pairing, tend to be inconvenient to conduct multiple additions since the synchronization of several streams of droplets becomes necessary but technically challenging [41]. Additionally, the electric field or chemicals used to induce coalescence may not be biocompatible.

In contrast to the paired droplet methods, a T-junction can be used to introduce a reagent as a continuous stream. The reagent is injected directly from the side channel into the droplets that flow in the main stream when they pass the T-junction [61,63,64]. In this approach, the reagent stream needs to flow much slower than the main stream to avoid the formation of new reagent only droplets within the main stream. To mitigate excess reagent flow, several methods are available to increase the efficiency of the direct injection method, such as the addition of inert gas spacers or a double T-junction structure. For instance, Nightingale et al. [61] used argon gas plugs to maintain uniform droplet spacing and mitigate the undesired formation of reagent only droplets. They employed the improved direct injection approach to conduct a five-stage quantum dot synthesis wherein particle growth was sustained by repeatedly adding fresh feedstock [61] as seen in Figure 2E.

One of the limitations of direct injection is the incompetency of injecting reagent into stable emulsions since the surfactants that are used to stabilize the target droplets prevent reagents from entering them. By leveraging electrocoalescence, Abate et al. [41] developed a picoinjector which used a pressurized channel to inject a controlled volume of reagent into preformed, PFPE-PEG-stabilized droplets at kHz frequency, as shown in Figure 2F. The injection volume can be controlled with sub-picoliter precision by tuning the droplet velocity and injection pressure. More importantly, they established serial and combinatorial injections by laying out several picoinjectors sequentially, each separately controlled by an electric field [41].

### 2.3. Flow Cytometric Sorting of Droplets

Commercially available fluorescent activated cell sorters are designed to interrogate suspended cells at ~10^7^ per hour and select the “hits” from a heterogeneous mixture based on the specific fluorescent and light scattering properties of the sample. FACS is, however, incompatible with non-aqueous carrier fluids. Thus, double emulsification of water-in-oil-in-water and other techniques become a plausible approach to enable the flow cytometric sorting of droplets, providing FACS compatible compartments to maintain the genotype-phenotype linkage in an aqueous carrier stream.

**Figure 3:**
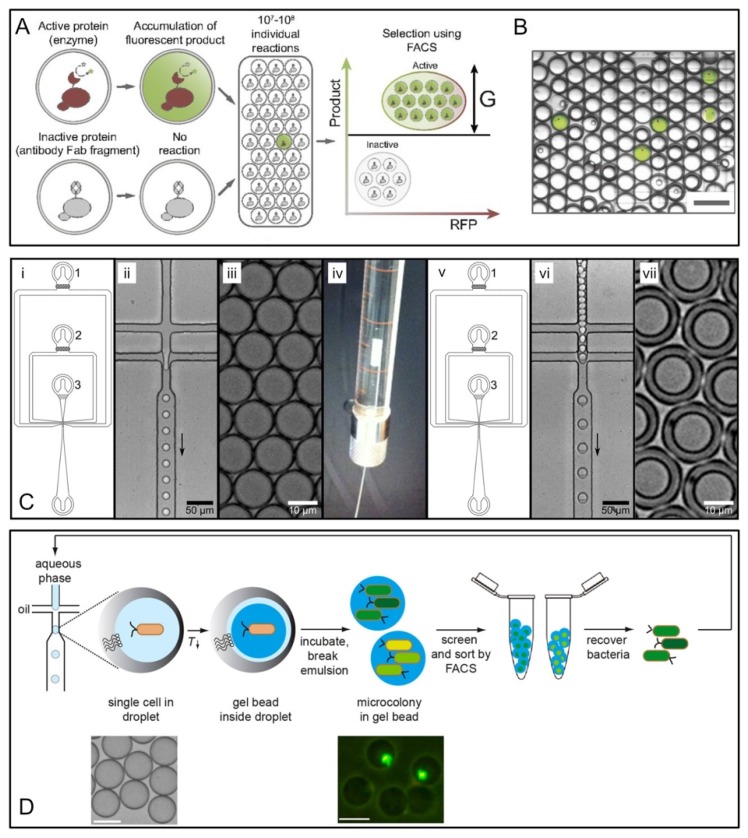
Flow cytometric sorting workflow using either double emulsion or hydrogel encapsulation. (**A**,**B**) Screening of biocatalysts anchored to the yeast surface using FACS on water-in-oil-in-water emulsions. (**A**) Compartmentalization of active and inactive yeast cells with fluorogenic substrate. After the mixture of active and inactive cells was encapsulated, the fluorescent product accumulated solely inside the droplets with active cells, which were selected using FACS. (**B**) Visualization of biochemical reaction by merging the signals of green fluorescence (reaction product), red fluorescence (reporter protein), and the visible light image. (Scale bar, 100 μm.) Figure 3A,B were adapted from Reference [29], complying with the License for PNAS Articles. (**C**) Formation of double emulsion droplets using a two-chip system. (i) Design of the device for primary emulsification; (ii) The aqueous samples are first mixed, then primary droplets are formed in the flow-focusing junction; (iii) Image of the monodisperse water-in-oil droplets; (iv) The emulsion droplets are taken up in a syringe, overlaid with mineral oil, and cushioned with a bottom layer of fluorinated oil; (v) A device with identical design to the first emulsification device, but a hydrophilic coating is used for formation of secondary emulsions; (vi) Image showing the production of water-in-oil-in-water double emulsion; (vii) Image of the monodisperse double emulsion droplets. Figure 3C was adapted from reference [21] under the Creative Commons Attribution license. (**D**) Overview of the FACS screening method with hydrogel encapsulation. Single cells are encapsulated into monodisperse water-in-oil emulsion droplets. The aqueous solution contains agarose that gels upon cooling on ice. After the formation of monoclonal microcolonies inside the beads, the beads are recovered from the emulsion and sorted by FACS. The bacteria are recovered from the gel beads and are then ready for a further round of analysis. Bottom left: a phase contrast microscope image of droplets (bottom left). Bottom right: a fluorescence microscope image of beads with two of them containing a microcolony. Scale bars: 50 μm. Figure 3D was adapted from reference [46] with permission from the American Chemical Society.

In vitro compartmentalization in double emulsions for FACS was demonstrated based on fluorescent signals in early 2000s [65,66,67]. However, double emulsions used in these pioneer studies were generated by homogenization with high polydispersity, which potentially limited the sensitivity and the throughput of FACS [29]. Recently, Terekhov et al. [29] combined microfluidic production of double emulsions with efficient FACS selection, as shown in Figure 3A,B. With integration with next-generation sequencing and liquid chromatography-mass spectrometry, their study provided deep insights into the genotype-phenotype linkages of the secretomes of encapsulated organisms. The functionality and versatility of the platform were demonstrated with the selection of different biocatalytic activities, screening enzymes with different levels of the same activity, de novo creation of enzymes with artificial activity, and investigation of bacterial cell-to-cell interactions [29].

Zinchenko et al. [21] also established a protocol with complex elements for quantitative analysis and sorting of monodisperse double emulsion droplets in a commercial flow cytometer. Their workflow (Figure 3C) incorporated additional steps, including assaying heat inactivation of lysates within the droplets, concentration of the encapsulated contents induced by droplet osmosis, and storage of droplets at −80 °C for discontinuous workflows. In their study, single *E. coli* cells expressing either wild-type arylsulfatase from *Pseudomonas aeruginosa* or a low activity variant (H211A) were initially co-compartmentalized with lysis reagents and enzyme substrate in water-in-oil droplets forming a primary emulsion (Figure 3Ci–iii). The primary emulsion droplets were then introduced into a second microfluidic device for double emulsification (Figure 3Civ–vii). The employment of two separate devices to produce double emulsion droplets rendered the independent size control of primary and double emulsions and the flexibility of surface coating (i.e., fluorophilic coating for primary emulsion and hydrophilic coating for double emulsion) [21]. The double emulsion droplets of interest contained active enzyme that was expressed by transformed *E. coli* and released due to cell lysis. The active enzyme hydrolyzed the substrate fluorescein disulfate and generated a fluorescent readout of enzyme activity. The droplets containing H211A variant only showed a low level of background fluorescence (due to the lack of hydrolytic activity). Finally, the highly fluorescent population was sorted via FACS to obtain the active variants. The results demonstrated that a sample with 0.01% active cells in the initial population was enriched 2500-fold. Further, 100,000-fold enrichment was achieved for another sample that initially contained only one hit in 1,000,000 cells (0.0001% cells expressing active enzyme), although with a higher droplet occupancy [21].

Eun et al. [57] described an HTS method for isolating spontaneous mutants of *E. coli* that had developed resistance to the antibiotic rifampicin using FACS. Instead of being compartmentalized into double emulsions, *E. coli* cells (MG1655-p*tet*EGFP) were encapsulated in agarose microparticles on a flow-focusing microfluidic device and incubated in the presence of varying concentrations of rifampicin. Microparticles containing Green Fluorescent Protein (GFP)-positive cells (0.2% of the total population) were sorted on FACS. The mutants were recovered from the microparticles and sequenced. As a result, an A1538T base-pair mutation was identified in the *rpoB* open reading frame. This mutation resulted in a Q513L substitution, conferring resistance to rifampicin. The overall screening consumed a total of 65 μg of rifampicin and took 6 h to complete, compared to 15 mg of compound that would have been required for a 48-h screening based on agar plates [57].

By performing FACS to sort cell-laden hydrogel microparticles, Duarte et al. [46] presented a high-throughput screening method to address challenges like substantial cell-to-cell variability and the requirement to check multiple states in synthetic biology (Figure 3D). In their study, single *E. coli* cells, either expressing or not expressing superfolder GFP, were encapsulated in water-in-oil droplets (20 μm in diameter), in which the aqueous phase, containing 1% agarose, solidified to a gel when chilled. FACS selection was performed after sufficient time to allow formation of microcolonies and expression of GFP. Sorted cells were recovered by enzymatic digestion of the agarose and plated on agar plates to determine the enrichment rate. The results showed that an enrichment of 30,000-fold was achieved for a 1:100,000 dilution (0.001%) of GFP expressing versus non-GFP expressing cells, which is comparable to enrichment rates previously reported [21,29,68].

Hydrogels are cross-linked hydrophilic polymers swollen in an aqueous environment [69]. Solute transport within hydrogels occurs primarily in the water-filled regions delineated by the polymer chains [69]. Therefore, cell-laden hydrogel microparticles may not be sufficient to prevent small molecules like substrates from diffusing between microparticles. However, the tunability of the physical and chemical parameters of hydrogels offers the opportunity of regulating the diffusion of solutes between microparticles in a selective manner. In other words, the structure and density of the crosslinking agent used in the formulation will affect the network mesh size, polymer chain mobility and the charge of polymer chains, and thus the rate of the diffusion of water-soluble molecules within and out of the hydrogel matrix [70]. Additionally, due to their mechanical properties, hydrogel particles can tolerate more shear and experience less deformation while they travel through microscale channels, thereby mitigating accidental rupture and coalescence. It has also been demonstrated with mammalian cells that oxidative stress was reduced by coating the cell-seeded gelatin core with a biodegradable silica shell without compromising the transport of nutrients to the cells encapsulated [71].

### 2.4. Fluorescence-Activated Droplet Sorting

Although in vitro compartmentalization can increase the versatility of FACS, flow cytometric sorting of double emulsions or hydrogel encapsulation still suffers from several limitations. For instance, the generation of double emulsions or hydrogel encapsulation needs additional steps of emulsification or microgel gelation/recovery, presenting additional complexity to the already lengthy droplet sorting process. It is also less convenient to manipulate the content of microcompartments like double emulsions after encapsulation.

**Figure 4:**
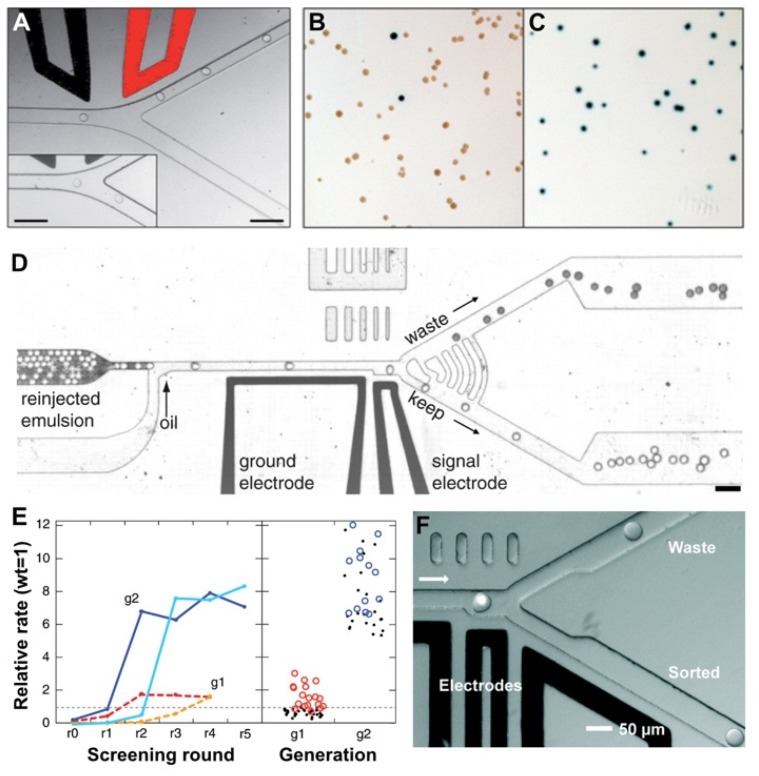
Fluorescence-activated droplet sorting systems. (**A**) Trajectories of droplets flowing through the sorting junction of a FADS system. When an AC electric field is applied across the electrodes, the droplets are deflected into the ‘positive’ arm. In the absence of the electric field, the droplets flowed into the ‘negative’ arm by default (inset). (**B**,**C**) Enrichment of cells by FADS based on β-galactosidase activity. Photographs of *E. coli* colonies before (B) and after sorting (C). The *lacZ* bacteria (blue colonies) were completely purified from the *ΔlacZ* bacteria (white colonies) after sorting, resulting in only *lacZ* colonies growing on the agar. Figure 3A–C were adapted from Reference [30] with permission from the Royal Society of Chemistry. (**D**) A different design of the FADS system. The droplets flow as a solid plug to a junction where oil is added to space the droplets. Light droplets contain 1 mM fluorescein and the dark ones contain 1% bromophenol blue. When a droplet passes by the laser that is focused on the channel at the gap between two electrodes, its fluorescence intensity is detected. If the intensity is above a threshold (in the case of light droplets), the droplet is sorted by dielectrophoresis towards the bottom channel. (**E**) Enrichment of library pools. The activities are normalized relative to wild-type HRP. The first-generation epPCR and saturation mutagenesis libraries (dashed red and orange, respectively) enrich to a level of about two times the activity of the wild type after four sorting rounds. The second-generation low- and high-mutation rate libraries (solid blue and cyan, respectively) enrich to about eight times the wild type. The right panel shows a dot plot of the activities of the 50 unique first-generation (g1) mutants (red circles) and 31 second-generation (g2) mutants (blue circles). Figure 3D,E were adapted from reference [19], complying with the License for PNAS Articles. (**F**) The sorting junction of another FADS system. Single droplet fluorescence was detected following excitation by the laser (the white dot). The default flow path of the droplet is towards the top ‘waste’ channel since the waste outlet is at atmospheric pressure and a withdrawal of less than half the total flow rate is applied to the ‘sorted’ outlet. However, if the droplet fluorescence exceeds a predefined threshold an electric field is activated between the electrodes, pulling the droplet to the bottom channel. Figure 3F was adapted from Reference [47] with permission from the Royal Society of Chemistry.

To address this challenge, Baret et al. [30] developed a fluorescence-activated droplet sorting (FADS) system that was optimized to directly sort picoliter-sized droplets by dielectrophoresis. Water-in-oil droplets were introduced into a microfluidic sorting device where they were spaced out and sorted at an asymmetric Y-shaped junction (Figure 4A–C). Droplets flowed along the wider ‘negative’ arm of the sorting junction by default due to the lower hydraulic resistance. If a droplet were to be sorted, based on a fluorescent signal for example, a pulse of high voltage alternating current would be applied across the electrodes adjacent to the sorting junction. The resulting electric field deflected the droplet of interest into the narrower ‘positive’ arm of the junction by dielectrophoresis [72]. To validate the system, *E. coli* cells, expressing either the reporter enzyme β-galactosidase or an inactive variant, were compartmentalized with fluorogenic substrates within 12-pL monodisperse droplets and sorted at a rate of ~300 droplets per second. The false positive (i.e., droplets that were sorted but contained inactive variant) error rate of the sorting device at this throughput was less than 1 in 10,000 droplets. When the cells were encapsulated at a lower density (e.g., ~1 cell for every 50 droplets), all of the sorted and recovered cells were the active strain. An even higher throughput (~2000 droplets per second) may be feasible but at the cost of an increase in the false positive error rate (e.g., 1 in 100 droplets).

Agresti et al. [19] explored the application of FADS in aiding the discovery of variants of the enzyme horseradish peroxidase (HRP) in directed evolution as shown in Figure 4D,E. Yeast cells (*Saccharomyces cerevisiae* EBY100) displaying enzyme variants on their surfaces were co-encapsulated into 6-pL aqueous droplets with a fluorogenic substrate. To ensure single-cell encapsulation and avoid confounding multiple cells, only ~22% droplets contained cells according to the Poisson distribution. As the droplets flowed through a FADS device, those containing the most active enzyme variants were sorted based on the fluorescence intensity. In each round of screening, the yeast cells were induced to express HRP, encapsulated into droplets and sorted at 2000 droplets per second for up to 3 h, with a total of up to 2 × 10^7^ cells being interrogated. As a result, new mutants of the enzyme HRP were identified exhibiting catalytic rates more than 10 times faster than their parent. In total, ∼10^8^ individual enzyme reactions were screened within 10 h, consuming less than 150 μL reagent. Compared with the state-of-the-art robotic screening systems, it was estimated that the FADS-based high-throughput screening system performed the entire assay with a 1000-fold increase in throughput and a 1-million-fold reduction assay reagent cost.

Sjostrom et al. [47] used a FADS system to screen a yeast library with mutations randomly introduced throughout the genome by UV-irradiation mutagenesis to select the cells with high α-amylase production (Figure 4F). A total of ~3 × 10^6^ droplets were sorted at a rate of 323 droplets per second over the course of slightly more than 2 h. They found that cells from the sorted subpopulation had over 60% higher enzyme production and 35% higher yield than the unsorted library. The top-performing clone was found to double the enzyme production compared with the mother strain. As suggested in this study, the platform had a throughput over 300 times higher than an automated microtiter plate screening system. At the same time, reagent consumption for a screening experiment was decreased by a million-fold, greatly reducing the costs of evolutionary engineering of production strains.

Similarly, Ma et al. [48] described a high-throughput microdroplet sorting system that could be used to screen up to ~10^7^ enzyme variants (10^8^ droplets) per day. The proposed system was able to evaluate two reaction channels simultaneously by adopting a dual-fluorescence detection/sorting microfluidic device. The employment of different combinations of two-color fluorogenic substrates enabled the screening for enzyme variants that had both improved catalytic activity and an additional enzymatic property such as regioselectivity, chemoselectivity, or enantioselectivity. As an example, Ma et al. [48] used the system to engineer the enantioselectivity of an esterase to preferentially produce desired enantiomers of profens, an important class of anti-inflammatory drugs. Using two types of selection modes over the course of five rounds of directed evolution, they identified a variant with 700-fold improved enantioselectivity for the desired (*S*)-profens from 5 million mutants.

### 2.5. Fluorescence-Activated Electrocoalescence

**Figure 5:**
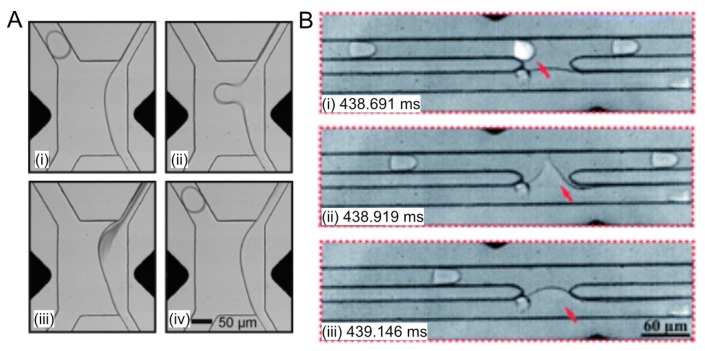
Fluorescence-activated electrocoalescence. (**A**) Selective extraction of the content of droplets of interest. (i) Below the threshold voltage, the electric field is insufficient to induce coalescence. As a result, the droplet passes by without coalescence. (ii and iii) When an additional square pulse is applied, an individual droplet is selected, and its contents are incorporated to the lateral aqueous stream. (iv) The applied voltage returns to its original value before the next droplet enters the electrode region and therefore the droplet flows past without coalescence. Figure 4A was adapted from Reference [73] with permission from John Wiley & Sons, Inc. (**B**) The fluorescence-activated electrocoalescence of a single droplet (indicated by the red arrow) in another FAE device. Figure 4B was adapted from Reference [49] with permission from the Royal Society of Chemistry.

Instead of sorting intact droplets containing “hits”, Fidalgo and coworkers [73] presented a different approach to selectively incorporate the contents of droplets initially flowing in a carrier oil phase into a continuous aqueous stream by electrocoalescence (Figure 5A). As soon as the fluorescence intensity of a droplet met a predefined criterion, an electric field across the channel was triggered to force the droplet of interest to cross the oil-water interface and coalesce with an aqueous stream at a frequency of 10–250 Hz. In their study, a mixture of fluorinated oil (FC-77) containing 30% (v/v) perfluorooctanol was employed as the carrier phase to compromise droplet stability and facilitate coalescence. Fluorescence-activated electrocoalescence has great potential of integrating with existing microfluidic modules for droplet-based screening. Additionally, the composition of the receptor stream can be adjusted to suit specific applications, such as quenching reactions to reach well-determined endpoints, providing favorable culture conditions for extracted cells, and lysing cells for downstream analysis [73]. However, the sorting method is destructive to the droplets, the content of the selected droplets is released into the aqueous stream via coalescence and the sorted droplets no longer exist.

The above electrocoalescence-facilitated droplet ‘sorting’ method was further optimized by Fallah-Araghi et al. [49] to achieve reliable sorting of up to 2000 droplets per second. In the design shown in Figure 5B, the gap in the coalescence section was minimized to allow efficient droplet extraction while maintaining a stable interface between the aqueous stream and the oil stream in the absence of an electrical field. They assembled the modules of encapsulation, droplet fusion, size-based droplet sorting and fluorescence-activated electrocoalescence to perform in vitro ultrahigh-throughput screening for protein engineering and directed evolution. Single genes were compartmentalized in aqueous droplets. After amplification by PCR, the droplets containing 30,000 copies of each gene were paired and fused with droplets containing a cell-free coupled transcription-translation (IVTT) system and the reagents for a fluorogenic assay. Then, genes from droplets containing desired activities were selectively recovered using fluorescence-activated electrocoalescence. As a result, a 502-fold enrichment was achieved from a mixture of *lacZ* genes encoding β-galactosidase and *lacZmut* genes encoding an inactive variant (molar ratio 1:100). Meanwhile, the volume and cost of PCR and IVTT reagents were reduced by ~105-fold compared with microtiter plate-based methods, achieving the screening of 10^6^ genes at the cost of 150 μL of reagents. In summary, we have compared the key advantages and disadvantages of the three main droplet sorting strategies in Table 2.

### 2.6. Mitigating Molecular Diffusion between Droplets

For droplets to be self-contained, a variety of surfactants have been explored to stabilize the water-oil interface and prevent droplet coalescence. Unfortunately, it has been found that many of the commonly used surfactants can actually assist molecular diffusion between droplets, resulting in cross-contamination of small, hydrophobic fluorescent molecules [39,58,59,74,75]. The leakage of fluorescent probes across droplet interface can be attributed to several phenomena. Fluorescent molecules may directly diffuse into the continuous phase [55,59]. Alternatively, micelles formed by surfactant molecules may facilitate the leakage of fluorescent probes [39,59]. In the latter case, a surfactant concentration below the critical micelle concentration would help mitigate the leakage but the droplets would become prone to coalescence if a low concentration of surfactants is used [55].

Since high-sensitivity assays are typically based on fluorescent readout, fluorophore exchange among aqueous droplets must be avoided to reliably compartmentalize the genotype-phenotype linkage. Courtois et al. [59] found that the addition of 5% (w/v) bovine serum albumin (BSA) significantly reduced the leakage of 3-*O*-methylfluorescein to 3%, compared with 45% for mineral oil containing 0.75% (w/w) Abil EM 90 surfactant. Moreover, Sandoz et al. [58] reported that the addition of 25% sucrose mitigated the leakage of the dye resorufin when mineral oil was mixed with different concentrations of surfactants combining 9% (w/w) Span 80 and 1% (w/w) TWEEN 80. They suggested that the sucrose strongly interfered with the surfactant’s ability to form micelles in water.

In fact, emulsion stability does not necessarily require amphiphilic surfactants to reduce the interfacial tension. The original observations by Ramsden [76] and the seminal work by Pickering [77] demonstrated that emulsion stability could be efficiently promoted by dispersed particles in the colloidal size range, which is now known as a Pickering emulsion. Prior work on making nanoparticle-stabilized emulsions in microfluidic systems mostly used hydrocarbons as the continuous phase [78,79,80]. Pan et al. [55], however, designed and synthesized silica nanoparticles (F-SiO_2_ NPs) and generated a Pickering emulsion in fluorinated oil. These nanoparticles are irreversibly adsorbed to the water-oil interface and do not form surfactant micelles which are likely responsible for the leakage of small molecules among aqueous droplets. In their study, the leakage of resorufin was prevented when the droplets were stabilized by F-SiO_2_ NPs. However, enzymes and proteins that adsorb nonspecifically to silica surfaces can become denatured. Thus, fluorinated silica nanoparticles could reduce enzyme activities and compromise the performance of droplet-based enzymatic assays [81,82]. To address this issue, the above Pickering emulsion system was improved by introducing PEG into the dispersed phase such that PEG could adsorb onto the surface of the F-SiO_2_ NPs from within the droplets [54]. The adsorption of PEG is driven by the formation of hydrogen bonds between PEG and the silanol groups on the nanoparticle surfaces [83]. PEG-adsorbed F-SiO_2_ NPs were found to be effective both in preserving enzyme activity and in preventing the leakage of small molecules. Although NPs are usually attached to the droplet surface more strongly than traditional surfactants due to the high desorption energy of NPs from the droplet surface, Pan et al. [84] showed that methods like electrocoalescence and perfluorooctanol addition worked well for merging NP-stabilized droplets with high efficiency to recover the contents of Pickering emulsions.

Instead of inhibiting molecular diffusion chemically, Siltanen et al. [85] constructed defined reactions with chemicals and cells incubated under air on an open array, thereby physically isolating the droplets and retaining hydrophobic compounds in their compartmentalized reactors. This oil-free picodrop bioassay platform was built upon the so-called Printed Droplet Microfluidics (PDM) technology that was developed by the same group in 2017 [86]. The ordered array of droplets generated by PDM functions analogously to microliter well plates—but at one-thousandth the scale—and uses a “microfluidic robot” for deterministic and programmable reagent and cell dispensing [86]. The PDM platform was used to detect the small differences in *trans*-β-farnesene production between similar strains of yeast. Farnesene is a sesquiterpene of the mevalonate and deoxyxylulose-5-phosphate pathways and readily soluble in emulsion oil. By compartmentalizing reactions under air, farnesene molecules remained partitioned with the cells responsible for their production, allowing direct measurements of product concentration for screening. The PDM platforms can interface with most bioanalytical tools and combines the throughput and low reagent consumption of droplet microfluidics with the flexibility and control of robotic fluid handling of physical arrays [85].

## 3. Commercialization and Commercial Activities of Droplet-based HTS in Protein Engineering

The protein engineering market is rapidly growing and promises lucrative opportunities given the increasing number of proteins and enzymes engineered for a multitude of applications in food, biopharmaceutical, and environmental industries. In fact, the commercialization of engineered proteins has manifested in economically beneficial and viable solutions for industry and healthcare sector. Protein engineering has also evolved to become a powerful tool contributing significantly to the developments in synthetic biology and metabolic engineering [87]. According to the report published by Zion Market Research in early 2019 [88], the continued expansion of the global protein engineering market is likely to be majorly driven by continuously developing pharmaceutical industries, biotechnological innovations, increasing focus toward targeting specific drug development, rising clinical and analytical techniques, and a substantial increase in research funding over the estimated timeframe. The report suggests that the global protein engineering market was valued at approximately USD 1.1 billion in 2018 and is projected to generate around USD 2.6 billion by 2024, at a compound annual growth rate (CAGR) of about 15.5% between 2019 and 2024. However, the costly and technologically complex tools and systems required for protein engineering, dearth of skilled labor, and need for highly qualified researchers pose as a hinderance to the growth of this market.

These challenges can be addressed by leveraging emerging technologies such as advanced computational protein design using artificial intelligence and machine learning and novel high-throughput screening enabled by miniature technologies like microfluidics. Droplet-based HTS technology has the potential to save considerable assay time and reagent cost, especially when exploring large libraries such as those from directed evolution. Droplet generation and cell encapsulation can now be performed readily on commercial microchips provided by microfluidics companies like Fluigent and Dolomite. Further, there are an increasing number of biotech companies applying or developing droplet-based microfluidics to accelerate the screening and selection for protein engineering. In a pilot study, researchers led by Merck coupled droplet microfluidics with mass spectrometry to accelerate the screening of enzyme evolution libraries for transaminase [89]. Velabs Therapeutics, a spin-off company launched by the European Molecular Biology Laboratory (EMBL), has been pioneering the screening of antibodies with modulatory function on complex signaling proteins using a fully integrated droplet-based microfluidics screening platform for phenotypic assays. Their system incorporates reinjection, fusion and sorting of droplets on a single microchip [90]. Additionally, Biomillenia and Merck are collaborating on the development of a market-leading enzyme in Merck’s portfolio of industry-leading enzyme solutions by utilizing Biomillenia’s droplet microfluidics screening technology. In a collaboration with Soufflet Biotechnologies, Biomillenia’s droplet screening platform was also used to develop novel cellulolytic and proteolytic enzymes as additives to animal feed, resulting in enhanced growth and expanding the range of raw materials for feed formulations. In addition, Hooke Bio, an Irish biotech company, developed a droplet-based, cell-screening device called Enigma which is highly adaptable for a multitude of applications including combinatorial drug screening. The Enigma device utilizes water-in-oil-in-water emulsions to allow diffusion of nutrients across the oil membrane and removal of waste products, which enables long-term (>48 h) cell culture for certain disease models such as type 1 diabetes. Sphere Fluidics provides an integrated platform (Cyto-Mine) to streamline cell encapsulation, assay, and FADS-based droplet sorting to accelerate the screening of antibiotic-resistant bacteria [91], antibody discovery and cell line development.

## 4. Challenges and Opportunities Facing Droplet Microfluidics in Protein Engineering

The past decade has witnessed a growing number of droplet-based HTS systems that accelerated the “design-build-test” cycles in the rational design and/or directed evolution of proteins. These exciting advancements also provided compelling evidence that droplet microfluidics can facilitate automation and enable new assay modalities for high-throughput screening. Nevertheless, there are still several potential challenges that need to be addressed. These challenges also represent the opportunities for continued innovation of microfluidics with implications for protein engineering.

(1) Most FADS systems rely on a lower hydraulic resistance along the ‘negative’ arm of the sorting junction to prevent droplets from entering the ‘positive’ arm in the absence of dielectrophoretic forces. Such a delicate hydraulic balance is very sensitive to the fluctuation of flow rates at the sorting junction. Fluctuations of fluid dynamics can be caused by the pulsing of syringe pumps, droplet aggregates, and/or the accumulation of precipitates during lengthy screening applications [53]. Therefore, more robust mechanisms to regulate the default flow path of droplets would be highly useful, especially for scaled-up, industrial applications. For example, multiple electrode pairs can be arranged parallel to the channels to achieve highly reliable two-way sorting, largely independent of the relative flow rates in the channels downstream of the sorting junction [53].

(2) Current droplet sorting systems based on water-in-oil emulsions almost always perform binary sorting. High-throughput multiplexed sorting capabilities would permit droplet sorting systems to sort based on multiplex signals more in line with flow cytometry. By integrating binary sorters serially, the sorting of three different droplet populations can be achieved, although the throughput was limited to 2–3 droplets per second [53]. Another FADS system was developed to sort up to five different droplet populations simultaneously at rates of several hundreds of droplets per second [51]. Multiplexed FADS systems like these will have the potential of significantly expanding the scope of how droplet-based HTS can accelerate the iterations for protein engineering.

(3) Variability in the number of cells per droplet due to stochastic cell loading is a major barrier to the encapsulation of cells within picoliter-sized monodisperse droplets. Dictated by the Poisson statistics, very low average loading densities have to be used to ensure single-cell encapsulation and avoid confounding multiple cells, which means that most droplets actually contain no cells and these ‘meaningless’ droplets consume the majority of the screening capacity. Approaches that can increase the single-cell occupancy can significantly increase the efficiency of droplet-based screening. Previously, inertial focusing was employed to evenly space cells as they travel rapidly within a high aspect ratio microchannel such that cells could enter the flow-focusing nozzle with the same frequency as droplet formation [92,93]. In the proof-of-concept experiment, over 80% droplets contained a single particle (i.e., 9.9 μm-diameter polystyrene beads), compared with about 40% from the Poisson distribution. Unfortunately, the technology of inertial focusing has been largely limited to bioparticles larger than red blood cells due to the strong correlation between the inertial lift forces and the particle size [94]. Very recently, Cruz et al. [95] has demonstrated inertial focusing in curved channels and the alignment of particles with diameters of 0.5–2 μm, a range that comprises a multitude of bacteria and yeast cells, which could be integrated with existing droplet-based HTS assays for protein engineering.

(4) Current microfluidic droplet sorting systems trigger the dielectrophoretic force based on fluorescence intensity, which renders these techniques high sensitivity and high throughput. However, fluorescence intensity can be biased by many factors such as background fluorescence, the dye concentration, the intensity of the light source, quenching and light scattering [56]. Thus, it would be highly desirable to explore droplet sorting systems, either active or passive, that are based on other properties such as average fluorescence lifetimes [56], luminescence, absorbance and droplet morphology [52]. For example, there has been a passive, size-based droplet separation device using deterministic lateral displacement (DLD), which takes advantage of the shrinkage of yeast-encapsulating droplets induced by the water efflux from these droplets to unoccupied droplets due to cell metabolism [52]. Hasan et al. [56] developed a droplet sorting approach based on the average fluorescence lifetimes of individual droplets. At a frequency of 40–50 Hz, they reliably sorted droplets containing either or both of the two dyes that were distinguishable by the average fluorescence lifetime [56].

(5) Another limitation of current droplet-based screening technologies is the requirement of a fluorogenic assay, which is not always available for many phenotypes. Gene expression profiling by mRNA sequencing is an alternative route for characterizing cell phenotypes. The emerging single-cell RNA sequencing droplet microfluidics (Drop-seq [96] and InDrop [97]) has the potential of being adapted for protein engineering in which the cell factories are usually bacterial and yeast cells that are much smaller than mammalian cells. In a pioneering study, Liu et al. [98] developed an isogenic colony sequencing (ICO-seq) system which integrated the expansion of yeast colonies in hydrogel microspheres with barcoded Drop-seq for high-throughput RNA sequencing, which can be used to characterize cellular phenotype for high-throughput screening applications.

(6) The capacity of droplet-based screening methods can be further boosted by leveraging the rapidly evolving technologies of artificial intelligence (AI), especially machine learning. Machine learning offers a route to enable automated monitoring of microfluidic systems by converting routinely collected sensor and image data into actionable information in real time [99]. It has been demonstrated very recently how machine learning-assisted image analysis can facilitate quality control over droplet generation [100] and efficiently code droplet populations [101]. With machine learning-supported image analysis, experimental conditions in microfluidic droplet assays can be encoded and decoded by colored beads [101]. Although it was not performed on droplets, a deep learning-assisted, image-activated cell sorting system was developed to sort in real time microalgal and blood cells based on intracellular protein localization and cell–cell interaction from large heterogeneous population [102].

## Figures and Tables

**Table 1:** Oil/surfactant formulations used in the studies that are discussed in this review.

Oil/Surfactant	Applications
HFE-7500/PFPE-PEG	Double emulsion for flow cytometric sorting [21], bacterial microcolonies in gel beads [46], FADS [30,31,47,48], electrocoalescence [49], triple emulsification for reagent addition [50], chemically-induced droplet coalescence (PFB added to induce coalescence) [45], picoinjector [41], multiplexed FADS [51], droplet size-based separation [52]
FC-40/PFPE-PEG	Double emulsion for flow cytometric sorting [21], FADS [19]
HFE-7500/QX200 (Bio-Rad proprietary oil)	2-way or 4-way FADS [53]
HFE-7500 or FC-40/fluorinated silica nanoparticles	Pickering emulsification to mitigate molecular diffusion [54,55]
Perfluorodecalin and perfluorooctanol (7:3 (v/v))	Fluorescence lifetime-activated droplet sorting [56]
Mineral oil/Span 80	Encapsulation of bacteria in agarose microparticles [57], emulsification of Amplex Ultra Red and horseradish peroxidase (specific sugars added to mitigate molecular diffusion) [58]
Mineral oil/Abil EM 90	Emulsification of 3-*O*-methylfluorescein (BSA added to mitigate molecular diffusion) [59]

**Table 2:** Advantages and disadvantages of existing droplet-based HTS technologies.

Droplet Sorting Mechanism	Advantages	Disadvantages
Flow cytometric sorting	Compatible with commercial instruments	Needs additional emulsification or hydrogel gelation; Droplet size is restricted by instrument specification
Fluorescence-activated droplet sorting (FADS)	Single emulsification; Sorted droplets are available for more rounds of sorting; Wide range of droplet sizes	Requires careful balance of “collection” and “waste” fluid flows; Needs additional step to recover the content inside the droplets
Fluorescence-activated electrocoalescence	Direct recovery of droplet content; Tunable conditions of the collection aqueous stream to perform additional chemistry	Sorted droplets no longer exist; Not possible to perform second round of sorting
